# Predictive Value of the D-Dimer-to-Fibrinogen Ratio for Acute Kidney Injury after Living-Donor Liver Transplantation: A Retrospective Observational Cohort Study Using Logistic Regression and Propensity Score Matching Analyses

**DOI:** 10.3390/jcm13185499

**Published:** 2024-09-17

**Authors:** Jaesik Park, Minju Kim, Jong-Woan Kim, Ho Joong Choi, Sang Hyun Hong

**Affiliations:** 1 Department of Anesthesiology and Pain Medicine, Seoul St. Mary’s Hospital, College of Medicine, The Catholic University of Korea, 222 Banpo-daero, Seocho-gu, Seoul 06591, Republic of Korea; 2 Department of Surgery, Seoul St. Mary’s Hospital, College of Medicine, The Catholic University of Korea, 222 Banpo-daero, Seocho-gu, Seoul 06591, Republic of Korea; hopej0126@gmail.com

**Keywords:** acute kidney injury, D-dimer-to-fibrinogen ratio, liver transplantation

## Abstract

**Background/Objectives:** Liver transplantation (LT) is typically performed as a surgery to treat end-stage liver disease (ESLD). Factors influencing acute kidney injury (AKI) post-living-donor LT (LDLT) have been identified; however, the potential role of the D-dimer-to-fibrinogen ratio (DFR) in predicting AKI remains unexplored. Therefore, we analyzed the relationship between DFR levels and the occurrence of AKI following LT. **Methods:** We retrospectively analyzed 648 recipients after 76 were excluded based on the exclusion criteria. Multivariate logistic regression and propensity score (PS) matching analyses were performed to evaluate the association between a high DFR (>1.05) and AKI. **Results:** After LDLT, AKI was observed in 148 patients (22.8%). A high DFR (>1.05) was independently associated with AKI. Multivariate logistic regression analysis showed that patients with a DFR above this threshold were four times more susceptible to AKI than those with a low DFR. A high DFR was also significantly associated with AKI in the propensity score-matched patients. **Conclusions:** Our findings suggest that incorporating preoperative DFR assessment into the management of patients undergoing LDLT could enhance the risk stratification for postoperative AKI.

## 1. Introduction

Liver transplantation (LT) is a definitive surgery for hepatic decompensation, and its incidence is increasing annually [1]. The prevalence of postoperative morbidity is high in patients who undergo LT, necessitating early intervention to optimize outcomes [2]. Acute kidney injury (AKI) is a frequent and significant adverse effect after LT [3,4,5], and various factors reportedly affect its occurrence [4]. Post-transplant AKI is often associated with high morbidity and mortality rates [6]; therefore, an early identification of its risk factors is crucial.

D-dimer, a byproduct of fibrin breakdown, is widely used to assess coagulation disorders, including pulmonary embolism (PE) and deep vein thrombosis (DVT). D-dimer formation reflects the development of fibrin-rich thrombus through the activation of the coagulation system [7]. Fibrinogen is a coagulation factor that acts as an indirect precursor of D-dimer in the fibrinolysis enzymatic cascade. D-dimer and fibrinogen have been extensively examined in several clinical contexts and are considered predictive markers for outcomes associated with systemic inflammation and infection [8,9,10]. In patients with liver cirrhosis, D-dimer levels tend to correlate with the severity of liver status [11]. In addition, patients with advanced liver cirrhosis show lower levels of fibrinogen than those with mild-to-moderate cirrhosis [12]. Low fibrinogen and high D-dimer levels with increased tissue plasminogen activator activity are due to hyperfibrinolysis, which frequently presents in patients with advanced liver disease [13,14]; therefore, we hypothesize that a high D-dimer-to-fibrinogen ratio (DFR) may be associated with the progression of liver disease. DFR has been investigated as a potential prognostic marker for PE or DVT [15,16]. Increasing evidence supports the use of DFR for predicting outcomes in severely ill patients [17,18]; however, the relationship between DFR and outcomes post-LT remains unclear. Previous studies have shown the association of D-dimer and fibrinogen with AKI after living-donor LT (LDLT) [19,20]. Therefore, we evaluated the prognostic utility of DFR for AKI in patients after LDLT and investigated its value in predicting postoperative morbidities.

## 2. Materials and Methods

### 2.1. Study Population

Here, 724 patients who received LT between March 2010 and February 2021 were reviewed retrospectively. The exclusion criteria included age <19 years, emergency procedures, deceased donor LT (DDLT), renal dysfunction (chronic kidney disease [21] and hepatorenal syndrome) and dialysis, and missing laboratory results. After excluding 76 patients, 648 were subsequently included in the final analysis. The Institutional Review Board approved this study (approval number: KC21RISI0576). Due to its retrospective design, informed consent was waived, and anonymized clinical data were used for analysis.

### 2.2. Liver Transplantation

The piggyback method, which preserves the recipient’s inferior vena cava, was applied in all patients. Hepatic vascular anastomoses were conducted on the Doppler ultrasound system to assess flow. General anesthesia was provided with appropriate hemodynamic management during the procedure. The use of blood components, including packed red blood cells (PRBCs), fresh frozen plasma (FFP), platelet concentrates (PCs), cryoprecipitate, and platelets, was based on laboratory or thromboelastography results [22]. Immunosuppressive medications, including Basiliximab, prednisolone, calcineurin inhibitors, and mycophenolate mofetil, were administered following the LDLT protocol of the hospital.

### 2.3. Measurements of D-Dimer and Fibrinogen

Blood samples were obtained via a sodium citrate tube preoperatively and D-dimer and fibrinogen were analyzed using an automated blood coagulation analyzer (CS-5100; Sysmex, Kobe, Japan). The DFR ratio was calculated as D-dimer (mg/L)/fibrinogen (mg/dL) × 100. The optimal cutoff of the DFR for the prediction of AKI development was determined using the area under the curve (AUC), and the optimal DFR ratio cutoff for the prediction of AKI development was 1.05 (AUC: 0.660; 95% confidence interval [CI]: 0.622–0.696; *p* < 0.001). Cutoffs for D-dimer (0.5 mg/L) and fibrinogen (160 mg/dL) were defined according to previous reports [23,24].

### 2.4. Preoperative and Intraoperative Findings

Preoperative findings included the Model for End-Stage Liver Disease (MELD) score, age, sex, body mass index (BMI), etiology, comorbidities (diabetes mellitus [DM] and hypertension), hepatic decompensation (ascites and varices), echocardiographic findings (diastolic dysfunction and ejection fraction), and laboratory parameters (glucose, potassium, hematocrit, albumin, international normalized ratio [INR], total bilirubin, DFR, ammonia, creatinine, sodium, calcium, white blood cells [WBC], and platelet count). Intraoperative findings included the presence of postreperfusion syndrome [25], intraoperative hemodynamic measurements (mean heart rate [HR], blood pressure, and central venous pressure [CVP]), blood component transfusion (PRBCs, FFP, PC, and cryoprecipitate), intraoperative hourly urine output, and fluid infusion indexed by body weight and blood loss. Graft findings involved graft fatty change, graft ischemic time, and the age and sex of donors.

### 2.5. Classification of Acute Kidney Injury

AKI was categorized as follows according to the Kidney Disease Improvement Global Outcomes classification [26]: Stage 1, increase in serum creatinine (sCr) level at least 0.3 mg/dL (within 48 h) or 1.5–1.9 times above the baseline sCr level (within 1 week); Stage 2, 2.0–2.9 times the baseline sCr value; ≥3.0 times the baseline sCr value; increase in sCr of at least 4.0 mg/dL; or renal replacement therapy irrespective of a previous stage.

### 2.6. Postoperative Outcomes

Postoperative outcomes included the duration of intensive care unit (ICU) stay, the occurrence of early allograft dysfunction (EAD), and death. EAD was identified based on one or more of the following: alanine transaminase or aspartate transaminase ≥2000 IU/mL within 7 days post-LT; an INR of at least 1.6 on postoperative day (POD) 7; and a total bilirubin of at least 10 mg/dL on POD 7 [27].

### 2.7. Statistical Analysis

Perioperative findings were analyzed using the Mann–Whitney *U*, χ^2^, or Fisher’s exact test. Potentially significant parameters (*p* < 0.1) in the univariate analysis were entered into the multivariate analysis. The AUC was used to evaluate predictive performance. Furthermore, 1:1 propensity score (PS) matching was utilized to balance the confounders in the high and low DFR groups [28]. PS matching was performed using a caliper with a width equal to 0.2 of the standard deviation of the logit of the propensity score. In variable selection, independent variables that are relevant to the study’s aim were selected. The variables were as follows: age, DM, BMI, sex, hypertension, ascites, varix, MELD score, albumin, ejection fraction, diastolic dysfunction, hematocrit, platelets, WBC count, glucose level, blood urea nitrogen, creatinine, total bilirubin, calcium, sodium, potassium, ammonia, INR, operation time, reperfusion syndrome, infusion of PRBC, FFP, and platelet concentrates, hourly urine output, mean CVP, blood pressure, HR, hourly fluid infusion, donor age, donor sex, donor-graft fatty change, total ischemic time, and graft recipient weight ratio. The correlation of inflammatory markers and length of ICU stay with the DFR was analyzed using Spearman’s rank correlation test. Delong’s methods were used to compare the AUC between AKI risk models. Statistical significance was set at *p* < 0.05. Statistical analyses were performed with SPSS Statistics Version 24.0 (SPSS Inc., Chicago, IL, USA) and MedCalc Statistical Software version 23.0.2 (MedCalc Software bv, Ostend, Belgium).

## 3. Results

### 3.1. Patient Characteristics

The patients enrolled in this study were predominantly men (77.2%). The median and interquartile ranges (IQRs) for the MELD score, BMI, and age were 13.6 (IQR: 6.4–23.6), 24.1 kg/m^2^ (IQR: 22–26.6 kg/m^2^), and 54 years (IQR: 48–60), respectively. The etiologies of end-stage liver disease were as follows: hepatitis B (55.9%), alcohol consumption (20.2%), hepatitis C (6.9%), autoimmune (4.3%), hepatitis A (4.2%), drugs and toxins (2%), and cryptogenic (6.5%).

### 3.2. Comparison of Perioperative Findings

Significant differences in preoperative recipient findings, including the MELD score, DM, ascites, and laboratory parameters, such as hematocrit, platelet count, albumin, total bilirubin, INR, and DFR, were identified in patients with and without AKI (Table 1). Furthermore, differences in intraoperative findings, including average HR, hourly urine output, total volumes of PRBCs, FFP, PC, and cryoprecipitate, intraoperative blood loss, and graft ischemic time, were identified (Table 2).

### 3.3. Association between Perioperative Findings and AKI Development

High DFR was independently associated with AKI, as well as the MELD score, DM, platelet count, hourly urine output, mean HR, cryoprecipitate transfusion, total ischemic time, and intraoperative blood loss in the multivariate logistic regression analysis (AUC: 0.740; 95% CI: 0.704–0.773; sensitivity: 75.68%; specificity: 63.0%; *p* < 0.001; Table 3). Patients with a high DFR (>1.05) had a four-times greater risk of AKI (odds ratio: 4.020; 95% CI: 2.230–7.247; *p* < 0.001).

### 3.4. Comparison of AUC between Logistic Models with DFR, D-Dimer, and Fibrinogen

**Figure 1:**
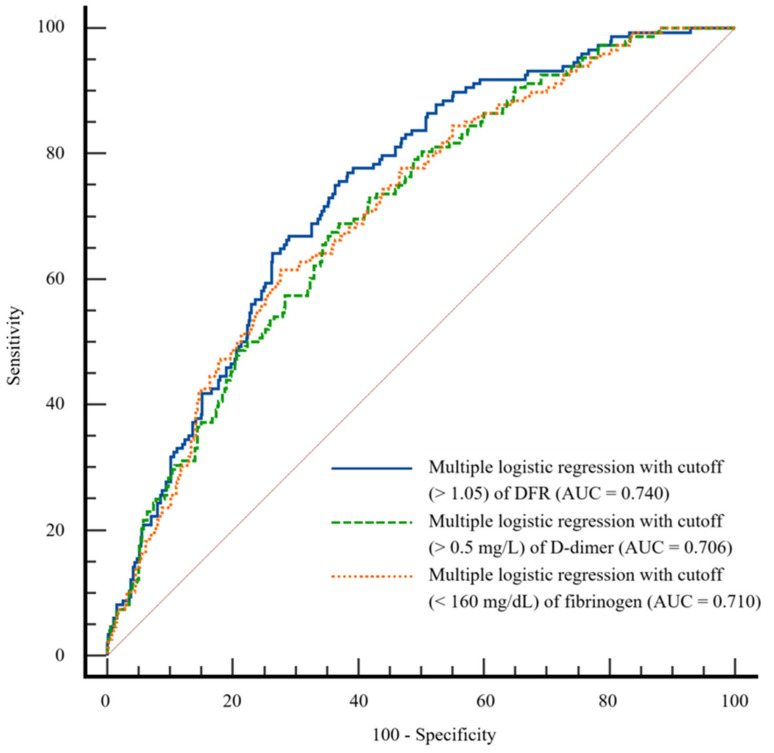
Comparison of area under the receiver operating characteristic curve for multiple logistic regression models with D-dimer-to-fibrinogen ratio (DFR), D-dimer, and fibrinogen.

We compared the AUCs for multiple logistic regression models with DFR, D-dimer, and fibrinogen (0.740, 0.706, and 0.710, respectively, Figure 1). According to Delong’s method, the predictive model with DFR showed a significantly better performance for AUC than the models with D-dimer and fibrinogen (*p* = 0.0071 and 0.0348, respectively).

### 3.5. Comparison of Predictive Accuracy of DFR, D-Dimer, and Fibrinogen for AKI Development

We compared the AUCs for AKI of a single variable of high DFR (>1.05), high D-dimer (>0.5 mg/L), and low fibrinogen (<160 mg/dL) (0.646, 0.584, and 0.591). According to Delong’s method, high DFR showed a significantly better performance for AUC than D-dimer and fibrinogen (*p* < 0.001 and 0.0145, respectively) (Table 4).

### 3.6. DFR Level and AKI Stage

**Figure 2:**
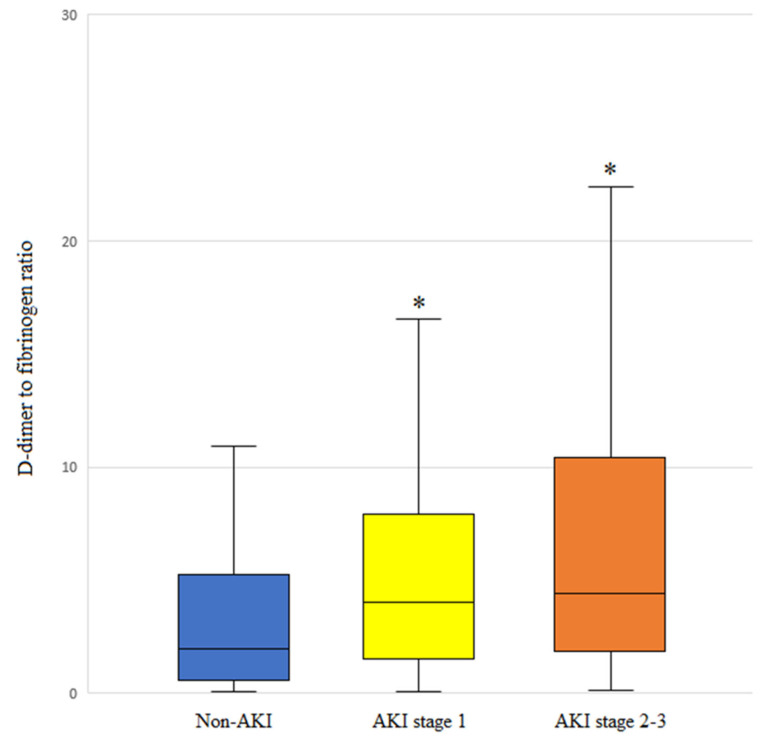
DFR levels based on the acute kidney injury (AKI) stage of patients who underwent living-donor liver transplantation. The box plots illustrate the median (line within the box), interquartile range (box), and 5th and 95th percentiles (whiskers). * *p* < 0.001 versus non-AKI.

DFR values were higher in patients with advanced-stage AKI (Figure 2). Notably, patients with non-AKI, AKI stage 1, and AKI stages 2–3 had a median (IQR) DFR of 1.7 (0.4–5.2), 4.0 (1.6–8.0), and 4.4 (1.9–10.4), respectively.

### 3.7. Association of Perioperative Findings with Severe AKI (AKI Stages 2–3)

In a multivariate logistic regression analysis for severe AKI (AKI stages 2–3), high DFR was independently associated with severe AKI, as well as hepatopulmonary syndrome, hepatocellular carcinoma, preoperative albumin, cryoprecipitate transfusion, BMI, oliguria, preoperative creatinine, the intraoperative mean albumin level, and intraoperative hematocrit (AUC: 0.810, CI: 0.778–0.840; sensitivity: 81.48%; specificity: 64.97%; *p* < 0.001).

### 3.8. Association of DFR with Inflammatory Factors

A significant correlation was identified between the DFR and inflammatory factors, including CRP (C-reactive protein), WBC, and albumin (all *p* < 0.001).

### 3.9. Association of High DFR with Postoperative Complications

A higher incidence of EAD, higher mortality rate, and longer ICU stay were observed among patients with a high DFR (*p* < 0.001, *p* = 0.008, and *p* = 0.005, respectively).

### 3.10. Perioperative Findings before and after PS Matching

Preoperative findings, such as albumin, ascites, MELD score, hematocrit, platelet count, WBC, blood urea nitrogen, creatinine, total bilirubin, sodium, ammonia, and INR, and intraoperative findings, such as PRBCs, FFP, PC, hourly urine output, and mean CVP, were statistically different between patients in the low and high DFR groups. After 1:1 nearest neighbor PS matching for 648 patients, 232 were successfully matched, and all standardized mean differences were <0.25 (Table 5). After PS matching, high DFR was significantly associated with AKI development (AUC: 0.662, CI: 0.597–0.772; sensitivity: 75.51%; specificity: 56.83%; *p* < 0.001).

### 3.11. Association between High DFR and AKI Occurrence in PS-Matched Patients

A high DFR was associated with AKI occurrence in the entire study cohort. After PS matching, high DFR remained a significant factor associated with AKI development (*p* < 0.001; Table 6).

### 3.12. Prevalence of AKI between the Low and High DFR Groups across Different AKI Stages in PS-Matched Patients

At each AKI stage, the occurrence of AKI was more frequent in the DFR group than in the low DFR group (*p* < 0.001; Table 7).

## 4. Discussion

Our findings suggest that high DFR (>1.05) is independently associated with a higher risk of AKI after LDLT and other factors such as the MELD score, DM, platelet count, intraoperative hourly urine output, mean HR, cryoprecipitate transfusion, total ischemic time, and blood loss. Patients with a high DFR had a four-fold greater risk of experiencing AKI than those with a low DFR (<1.05). The DFR significantly increased with AKI severity, and patients with a high DFR had worse outcomes regarding morbidity and mortality.

AKI is a prevalent complication after LT, occurring in 5–94% of patients [4]. Its etiology is not fully understood; however, potential contributing factors include hypovolemia, inflammation, and nephrotoxins [29,30]. Recent research has highlighted the critical role of systemic inflammation in the development of AKI [31]. Inflammatory cytokines interact with tubular epithelial cells and contribute to kidney damage [32,33]. AKI is a major risk factor that increases the likelihood of complications and mortality after LDLT [34]. Therefore, it is important to evaluate the risk factors for AKI in patients undergoing LDLT.

D-dimer is primarily produced during secondary fibrinolysis in thrombotic disorders [7]. Recent reports have suggested that higher levels of D-dimer are associated with a worse prognosis in critically ill patients [35,36], and D-dimer levels tend to correlate with the severity of liver status in patients with liver cirrhosis [9,37]. Fibrinogen levels can be elevated in acute-phase reactions, such as systemic inflammation or infection. However, its level tends to decline with an increasing severity of liver cirrhosis. These findings imply a close correlation between the severity of cirrhosis and altered hemostasis [38,39]. DFR may reflect the state of coagulation and fibrinolytic process, and elevated DFR might potentially represent a prothrombotic status [40,41]. The inverse relationship between D-dimer and fibrinogen implies that activated coagulation leads to fibrinogen consumption and concurrent fibrinolysis, resulting in elevated D-dimer levels [41]. Similarly, decompensated cirrhosis often exhibits hyperfibrinolysis, characterized by low fibrinogen levels, increased tissue plasminogen activator activity, and elevated D-dimer levels [13]. Therefore, high DFR, which indicates high D-dimer and low fibrinogen levels, might be associated with the progression of liver disease and associated hyperfibrinolysis [14].

In patients with liver cirrhosis, the progression of vasodilatation activates vasoconstrictive systems, and the activation of the renin–angiotensin system results in renal vasoconstriction and renal blood flow decrease [42]. Renal injury is more greatly facilitated by factors that cause a decrease in effective blood volume, such as excessive diuresis or bleeding and systemic inflammation. Therefore, as cirrhosis progresses, patients may become vulnerable to developing AKI, and AKI can occur due to massive blood loss during LT or systemic inflammation. Hyperfibrinolysis is associated with advanced liver cirrhosis; therefore, high DFR followed by LT is possibly associated with the occurrence of AKI [13,14]. Elevated D-dimer levels are associated with inflammatory coagulation and fibrinolysis, which could contribute to AKI development in critically ill patients [19,43]. A low fibrinogen concentration is reportedly associated with systemic inflammation in patients with liver cirrhosis [12]. DFR showed a significant association with inflammatory factors such as CRP, WBC, and albumin in the present study; therefore, the relationship between DFR and AKI may be affected by systemic inflammation. The etiology of cirrhosis could also be a factor responsible for AKI. In a previous report [44], the etiology of cirrhosis, such as alcoholic, metabolic-dysfunction-associated steatohepatitis, was a predictive factor for AKI. We evaluated alcoholic liver cirrhosis as a preoperative factor in the univariate logistic regression, and the *p*-value was 0.089 (Table 3); however, it was not included in the multivariate logistic model.

The intraoperative hourly urine output, DM, platelet count, mean HR, MELD score, cryoprecipitate transfusion, total ischemic time, and intraoperative blood loss were also associated with AKI in the multivariate logistic regression analysis. A reduced urine output may be associated with AKI in relation to impaired perfusion to the glomerular afferent arteries, considering that a decreased urine output often indicates hypotension or hypovolemia [45]. The precise mechanisms underlying AKI development in diabetic kidneys are not fully understood; however, a diminished resilience to reduced renal perfusion following ischemia or increased apoptosis of proximal tubular cells are potential mechanisms [46]. The MELD score, ischemic time, platelet count, HR, cryoprecipitate transfusion, and blood loss are well-known predictors of AKI in patients undergoing LT [3,4,5,47,48].

DFR has been suggested as a novel predictor of the prognosis in various conditions, including PE and COVID-19 infection [16,18]. DFR is reportedly a factor associated with survival in patients undergoing percutaneous coronary intervention [17]. In our study, the high DFR group had a higher incidence of EAD, longer ICU stays, and lower overall survival than the low DFR group. Therefore, DFR is thought to be a critical prognostic marker in LT recipients. DFR was also significantly associated with AKI in PS-matched patients. Considering that there was no statistical difference between the high and low DFR groups, high DFR may serve as an early indicator of a more advanced disease status and increased susceptibility to AKI.

This study has some limitations. First, we could not identify a specific mechanism linking high DFR to AKI development. High D-dimer and low fibrinogen levels are closely associated with advanced liver cirrhosis and inflammation; however, the precise pathways remain unclear. Second, the cutoff values for DFR used in this study differ from those used to diagnose coagulation disorders. Different clinical settings may require different cutoff values. Third, AUC values for the models for AKI development were 0.7–0.8, and this signifies moderate discrimination. According to the prediction models for AKI following LT [49], the predictive models with AUC > 0.8 were as follows: predictive model for severe AKI such as acute renal failure requiring renal replacement therapy or AKI 2–3; predictive model for AKI 1–3 with postoperative factor, relatively small dataset (<200), or machine learning analysis. The AUC for the predictive model for AKI 2–3 was also >0.8 in the present study; however, excluding postoperative factors may limit the AUC value. Fourth, owing to its retrospective design, this study is subject to inherent biases that could not be entirely controlled despite PS matching. Finally, the relationship between DFR and the occurrence of AKI might vary depending on the donor type. Therefore, further research is required to investigate the prognostic value of DFR after DDLT.

## 5. Conclusions

Considering that AKI is a prevalent concern in patients receiving LDLT and is closely associated with poor outcomes, identifying risk factors preoperatively and intraoperatively is crucial. Based on the findings of our study, a high preoperative DFR could be a promising indicator of AKI risk, providing valuable insights into the patient’s overall medical status. Therefore, integrating DFR with factors such as hourly urine output, mean HR, DM, platelet count, MELD score, cryoprecipitate transfusion, total ischemic time, and blood loss in a predictive model may enhance the ability to predict postoperative AKI. Risk factors for AKI development, including DFR, must be assessed preoperatively, and patients with these risk factors should be carefully monitored.

## Figures and Tables

**Table 1:** Preoperative recipient findings in the non-AKI and AKI groups.

Group	Non-AKI	AKI	*p*
n	500	148	
Age (years)	54 (48–60)	53 (48–59)	0.440
Sex (male)	341 (68.2%)	109 (73.6%)	0.206
Body mass index (kg/m^2^)	24 (22–26)	24 (22–28)	0.092
Etiology			
Alcohol	93 (18.6%)	38 (25.7%)	0.309
Hepatitis A	18 (3.6%)	9 (6.1%)	
Hepatitis B	284 (56.8%)	78 (52.7%)	
Hepatitis C	37 (7.4%)	8 (5.4%)	
Autoimmune	24 (4.8%)	4 (2.7%)	
Drugs and toxins	10 (2.2%)	3 (1.4%)	
Cryptogenic	33 (6.6%)	9 (6.1%)	
Comorbidity			
Diabetes mellitus	121 (24.2%)	49 (33.1%)	0.030
Hypertension	101 (20.2%)	31 (20.9%)	0.843
MELD score (point)	12 (6–23)	17 (9–25)	0.006
Hepatic decompensation			
Varix	114 (22.8%)	41 (27.7%)	0.219
Ascites	224 (44.88%)	84 (56.8%)	0.011
Cardiac function			
Ejection fraction (%)	64 (62–67)	64 (62–67)	0.289
Diastolic dysfunction	200 (40.0%)	65 (43.9%)	0.244
Laboratory variables			
Hematocrit (%)	30 (25–36)	28 (24–32)	0.004
WBC count (×10^9^/L)	4.4 (2.8–6.8)	4.5 (3.1–8.2)	0.335
Albumin (g/dL)	3.1 (2.7–3.6)	2.9 (2.6–3.3)	<0.001
Platelet count (×10^9^/L)	68 (47–109)	56 (39–76)	<0.001
International normalized ratio	1.4 (1.2–2.1)	1.6 (1.3–2.0)	0.037
DFR level	1.7 (0.4–5.3)	4.1 (1.6–9.2)	<0.001
Total bilirubin (mg/dL)	2.1 (0.7–11.7)	3.6 (1.3–17.1)	0.002
Sodium (mEq/L)	139 (135–142)	138 (134–141)	0.086
Potassium (mEq/L)	4 (3.7–4.3)	4 (3.5–4.3)	0.273
Calcium (mg/dL)	8.4 (8–8.9)	8.4 (7.9–8.7)	0.166
Glucose (mg/dL)	107 (92–138)	113 (95–146)	0.165
Creatinine (mg/dL)	0.9 (0.7–1.1)	0.9 (0.7–1.3)	0.299
Ammonia (μg/dL)	95 (64–147)	100 (67–156)	0.392

Abbreviations: AKI, acute kidney injury; DFR, D-dimer-to-fibrinogen ratio; MELD, Model for End-stage Liver Disease; WBC, white blood cell. NOTE: Values are medians (ranges) or numbers (percentages).

**Table 2:** Intraoperative recipient and donor-graft findings in the non-AKI and AKI groups.

Group	Non-AKI	AKI	*p*
n	500	148	
Surgical duration (min)	495 (440–560)	506 (456–584)	0.103
Postreperfusion syndrome	256 (51.2%)	87 (58.8%)	0.104
Average of vital signs			
MBP (mmHg)	75 (70–82)	76 (68–85)	0.520
HR (beats/min)	89 (80–99)	93 (83–102)	0.037
CVP (mmHg)	9 (7.3–11)	9.3 (7–11.5)	0.689
Blood product transfusion (unit)			
Packed red blood cells	7 (4–13)	10 (6–16)	<0.001
Fresh frozen plasma	6 (4–10)	10 (6–12)	<0.001
Platelet concentrate	4 (0–8)	6 (0–12)	0.009
Cryoprecipitate	0 (0–0)	0 (0–0)	<0.001
Blood loss (L)	2.9 (2.2–3.8)	3.1 (2.5–4.5)	0.004
Hourly fluid infusion (mL/kg/h)	10.5 (8.2–14.1)	11.1 (8.2–15)	0.273
Hourly urine output (mL/kg/h)	1.6 (0.8–2.4)	0.9 (0.6–1.6)	<0.001
Donor-graft finding			
Age (years)	35 (26–42)	35 (26–40)	0.751
Sex (male)	310 (62%)	93 (63%)	0.854
GRWR (%)	1.2 (1.0–1.5)	1.2 (1.1–1.5)	0.204
Graft ischemic time (min)	87 (68–105)	96 (73–106)	0.041
Fatty change (%)	5 (1–5)	4 (0–5)	0.596

Abbreviations: AKI, acute kidney injury; CVP, central venous pressure; GRWR, graft recipient weight ratio; HR, heart rate; MBP, mean blood pressure. NOTE: Values are medians (interquartile ranges) or numbers (percentages) unless indicated otherwise.

**Table 3:** Association of preoperative and intraoperative findings with the occurrence of AKI in patients who underwent living-donor liver transplantation.

	Univariate Analysis				Multivariate Analysis			
	*β*	Odds Ratio	95% CI	*p*	*β*	Odds Ratio	95% CI	*p*
Preoperative recipient factor								
Age (years)	−0.003	0.997	0.978–1.016	0.748				
Sex (male vs. female)	−0.265	0.767	0.509–1.158	0.207				
Body mass index (kg/m^2^)	0.044	1.045	0.998–1.093	0.061				
Comorbidity								
Diabetes mellitus	0.438	1.550	1.040–2.310	0.031	0.433	1.541	1.003–2.368	0.048
Hypertension	0.046	1.047	0.666–1.645	0.843				
Alcoholic liver cirrhosis	0.378	1.459	0.944–2.254	0.089				
MELD score (point)	0.018	1.018	1.001–1.035	0.033	−0.027	0.974	0.952–0.996	0.021
Hepatic decompensation								
Varix	0.260	1.297	0.856–1.967	0.220				
Ascites	0.481	1.617	1.117–2.341	0.011				
Cardiac function								
Ejection fraction (%)	0.029	1.029	0.988–1.073	0.173				
Diastolic dysfunction	0.161	1.175	0.811–1.702	0.395				
Laboratory variables								
Hematocrit (%)	−0.041	0.960	0.933–0.988	0.005				
WBC count (×10^9^/L)	0.014	1.015	0.985–1.046	0.318				
Albumin (g/dL)	−0.610	0.543	0.392–0.753	<0.001				
Platelet count (×10^9^/L)	−0.008	0.992	0.988–0.996	0.001	−0.005	0.995	0.991–1.000	0.033
International normalized ratio	0.094	1.098	0.885–1.363	0.396				
Total bilirubin	0.015	1.015	1.000–1.031	0.056				
High DFR (>1.05)	1.661	5.267	3.082–9.001	<0.001	1.391	4.020	2.230–7.247	<0.001
Comparative factors * High D-Dimer (>0.5 mg/L)	1.445	4.243	1.919–9.381	<0.001	0.834	2.302	0.999–5.303	0.050
Low fibrinogen (<160 mg/dL)	0.742	2.099	1.442–3.055	<0.001	0.402	1.494	0.988–2.260	0.057
Sodium (mEq/L)	−0.023	0.977	0.945–1.009	0.163				
Potassium (mEq/L)	−0.157	0.854	0.625–1.167	0.323				
Calcium (mg/dL)	−0.153	0.859	0.670–1.101	0.229				
Glucose (mg/dL)	0.001	1.001	0.998–1.005	0.408				
Creatinine (mg/dL)	−0.116	0.890	0.744–1.065	0.205				
Ammonia (μg/dL)	0.001	1.001	0.999–1.003	0.325				
Intraoperative recipient factor								
Surgical duration (min)	0.001	1.001	0.999–1.003	0.105				
Postreperfusion syndrome	0.307	1.359	0.938–1.971	0.105				
Average of vital signs								
MBP (mmHg)	0.002	1.002	0.996–1.008	0.468				
HR (beats/min)	0.014	1.015	1.001–1.028	0.031	0.013	1.013	0.999–1.027	0.069
CVP (mmHg)	0.023	1.024	0.969–1.082	0.404				
Blood product transfusion (unit)								
Packed red blood cells	0.032	1.032	1.012–1.053	0.001				
Fresh frozen plasma	0.032	1.0333	1.008–1.058	0.008				
Platelet concentrate	0.002	1.002	0.990–1.014	0.769				
Cryoprecipitate	0.156	1.169	1.081–1.264	<0.001	0.013	1.111	1.022–1.208	0.013
Blood loss (L)	0.107	1.113	1.017–1.218	0.020	0.054	1.055	0.986–1.129	0.121
Hourly fluid infusion (mL/kg/h)	0.012	1.012	0.995–1.030	0.159				
Hourly urine output (mL/kg/h)	−0.390	0.677	0.562–0.816	<0.001	−0.358	0.699	0.551–0.889	0.003
Donor-graft factor								
Age (years)	−0.005	0.995	0.979–1.012	0.586				
Sex (male)	−0.036	0.965	0.660–1.410	0.854				
GRWR (%)	0.179	1.196	0.836–1.710	0.327				
Graft ischemic time (min)	0.008	1.008	1.000–1.015	0.030	0.007	1.007	1.000–1.015	0.062
Fatty change (%)	0.008	1.006	0.981–1.035	0.572				

Abbreviations: CI, confidence interval; CVP, central venous pressure; DFR, D-dimer-to-fibrinogen ratio; GRWR, graft recipient weight ratio; HR, heart rate; MBP, mean blood pressure; MELD, Model for End-Stage Liver Disease; WBC, white blood cell. * Odds ratio of high D-dimer and low fibrinogen were values from other univariate and multivariate logistic regression models without DFR.

**Table 4:** Predictive accuracy of DFR, D-dimer, and fibrinogen for AKI development.

	AUC	95% CI	*p*
High FDR (>1.05)	0.646	0.607–0.682	<0.001
High D-Dimer (>0.5 mg/L)	0.584	0.545–0.622	<0.001
Low fibrinogen (<160 mg/dL)	0.591	0.552–0.630	<0.001

Abbreviations: AKI, acute kidney injury; DFR, D-dimer-to-fibrinogen ratio; PS, propensity score; CI, confidence interval.

**Table 5:** Comparison of perioperative findings between the high and low DFR groups using propensity score matching analysis.

Group	Before Propensity Score-Matched Analysis				After Propensity Score-Matched Analysis			
	High DFR	Low DFR	*p*	SD	High DFR	Low DFR	*p*	SD
n	428	220			116	116		
Preoperative finding								
Age (years)	53 (47–59)	55 (50–60)	0.005	−0.173	53 (49–60)	60 (54–64)	0.528	0.009
Sex (male)	281 (65.7%)	169 (76.8%)	0.003	−0.235	86 (74.1%)	79 (68.1%)	0.311	0.127
Body mass index (kg/m^2^)	24 (22–27)	24 (22–26)	0.215	0.150	24 (22–26)	24 (22–26)	0.723	−0.02
Diabetes mellitus	112 (26.2%)	58 (26.4%)	0.957	−0.004	33 (28.4%)	29 (25.0%)	0.553	0.078
Hypertension	74 (17.3%)	58 (26.4%)	0.007	−0.240	25 (21.6%)	24 (20.7%)	0.872	0.023
MELD	18 (10–27)	6 (4–12)	<0.001	0.918	13 (6–21)	11 (6–18)	0.351	0.023
Varix	118 (27.6%)	37 (16.8%)	0.002	0.240	25 (21.6%)	30 (25.9%)	0.440	−0.096
Ascites	259 (60.5%)	49 (22.3%)	<0.001	0.781	53 (45.7%)	46 (39.7%)	0.353	0.123
Ejection fraction	64 (62–67)	64 (62–66)	0.173	0.075	64 (63–67)	64 (62–66)	0.302	0.048
Diastolic dysfunction	176 (41.1%)	89 (40.5%)	0.870	0.014	44 (37.9%)	44 (37.9%)	1.000	<0.001
Laboratory variables								
Hematocrit (%)	27 (24–32)	34 (29–39)	<0.001	−0.878	30 (26–36)	30 (25–35)	0.517	0.102
White blood cell count (×10^9^/L)	4.8 (3.0–8.9)	4.0 (2.7–5.1)	<0.001	0.370	4.5 (2.8–8.0)	3.7 (2.5–5.8)	0.005	0.177
Albumin (g/dL)	2.9 (2.6–3.3)	3.4 (3.0–3.9)	<0.001	−0.890	3.1 (2.7–3.5)	3.1 (2.7–3.4)	0.775	0.047
Platelet count (×10^9^/L)	57 (41–82)	90 (60–132)	<0.001	−0.652	67 (46–109)	73 (49–103)	0.548	0.093
Sodium (mEq/L)	138 (134–141)	141 (139–142)	<0.001	−0.512	140 (136–142)	140 (136–142)	0.584	−0.062
Potassium (mEq/L)	4 (3.6–4.4)	4 (3.7–4.3)	0.898	0.041	4.0 (3.6–4.3)	4.0 (3.7–4.3)	0.955	0.049
Calcium (mEq/L)	8.4 (7.9–8.8)	8.5 (8.1–8.9)	0.002	−0.112	8.4 (8.0–8.8)	8.3 (7.9–8.8)	0.421	0.062
Glucose (mg/dL)	114 (93–148)	103 (91–126)	0.003	0.237	107 (92–138)	106 (93–139)	0.895	0.026
Urea nitrogen	18 (12–34)	13 (11–16)	<0.001	0.463	16 (10–25)	14 (11–17)	0.294	0.120
Creatinine (mg/dL)	0.9 (0.6–1.5)	0.8 (0.7–1.0)	0.015	0.251	0.8 (0.6–1.2)	0.8 (0.7–1.0)	0.774	0.096
Total bilirubin	4.4 (1.4–18.1)	1.0 (0.6–2.4)	<0.001	0.566	1.9 (0.9–7.1)	1.7 (0.9–6.8)	0.688	0.008
Ammonia	100 (68–162)	87 (62–136)	0.006	0.254	100 (65–152)	113 (66–162)	0.633	0.089
INR	1.7 (1.4–2.3)	1.2(1.1–1.4	<0.001	0.691	1.5 (1.2–1.9)	1.4 (1.2–1.6)	0.271	0.193
Intraoperative finding								
Total surgery duration (min)	503 (450–570)	490 (435–540)	0.094	0.078	505 (450–595)	500 (436–579)	0.428	0.089
Postreperfusion syndrome	236 (55.1%)	107 (48.6%)	0.116	0.131	57 (49.1%)	56 (48.3%)	0.895	0.017
Average vital signs								
MBP (mmHg)	76 (70–84)	75 (69–82)	0.434	0.094	75 (69–83)	77 (70–83)	0.512	−0.001
HR (beats/min)	91 (80–102)	88 (80–97)	0.031	0.160	91 (80–98)	87 (79–97)	0.212	0.164
CVP (mmHg)	9 (8–12)	9 (7–11)	0.001	0.295	9 (7–11)	9 (7–11)	0.734	0.085
Blood product transfusion (unit)								
Packed red blood cells	10 (6–16)	4 (2–8)	<0.001	0.698	9 (5–12)	5 (4–8)	0.004	0.169
Fresh frozen plasma	10 (6–13)	4 (3–6)	<0.001	0.757	8 (5–10)	6 (4–10)	<0.001	0.135
Platelet concentrate	6 (0–12)	0 (0–5)	<0.001	0.394	5 (0–10)	0 (0–6)	0.034	0.129
Hourly fluid infusion (mL/kg/h)	10.9 (8.1–15.2)	10.4 (8.3–13.4)	0.185	0.156	11.0 (7.7–14.3)	10.4 (7.9–14.0)	0.893	0.026
Hourly urine output (mL/kg/h)	1.07 (0.6–1.9)	1.9 (1.2–2.8)	<0.001	−0.806	1.4 (0.8–2.4)	1.6 (0.9–2.4)	0.557	−0.075
Donor-graft finding								
Age (years)	35 (26–41)	34 (26–44)	0.970	−0.032	35 (27–45)	34 (26–44)	0.866	0.013
Sex (male)	271 (63.3%)	132 (60.0%)	0.410	−0.069	72 (62.1%)	69 (59.5%)	0.687	−0.054
GRWR (%)	1.2 (1.0–1.5)	1.2 (1.1–1.6)	0.582	−0.039	1.3 (1.0–1.5)	1.2 (1.0–1.5)	0.725	−0.036
Graft ischemic time (min)	91 (69–107)	87 (69–100)	0.166	0.128	83 (66–100)	85 (69–100)	0.653	−0.049
Fatty change (%)	5 (1–5)	4 (0–5)	0.010	0.089	5 (1–5),	5 (1–5)	0.873	0.005

Abbreviations: DFR, D-dimer-to-fibrinogen ratio; MELD, Model for End-Stage Liver Disease; INR, international normalized ratio; MBP, mean blood pressure; HR, heart rate; CVP, central venous pressure; GRWR, graft recipient weight ratio. NOTE: Values are expressed as median (interquartile) and numbers (proportions).

**Table 6:** Association of high DFR with postoperative AKI in the entire study population and PS-matched patients.

	*ß*	Odds Ratio	95% CI	*p*
In the entire study population (n = 648)				
High FDR (vs. low FDR)	1.661	5.267	3.082–9.001	<0.001
In the PS-matched study population (n = 232)				
High FDR (vs. low FDR) adjusted for PS	1.401	4.059	1.988–8.288	<0.001

Abbreviations: AKI, acute kidney injury; DFR, D-dimer-to-fibrinogen ratio; PS, propensity score; CI, confidence interval.

**Table 7:** Comparison of the prevalence of AKI between the low and high DFR groups across different AKI stages in PS-matched patients.

Group	Low DFR (<1.05)	High DFR (>1.05)	*p*
n	116	116	<0.001
Non-AKI	104 (57.1%)	78 (42.9%)	
AKI stage 1	9 (25.0%)	27 (75.0%)	
AKI stages 2–3	3 (21.4%)	11 (78.6%)	

Abbreviations: AKI, acute kidney injury; DFR, D-dimer-to-fibrinogen ratio. NOTE: Values are expressed as numbers (with % proportion).

## Data Availability

The data presented in this study are available on reasonable request.

## Abbreviations

LT, liver transplantation; AKI, acute kidney injury; PE, pulmonary embolism, DVT, deep vein thrombosis, DFR, D-dimer-to-fibrinogen ratio; LDLT, living-donor liver transplantation; DDLT, deceased-donor liver transplantation; MBP, mean blood pressure; PRBC, packed red blood cells; FFP, fresh frozen plasma; PC, platelet concentrate; AUC, area under the curve; MELD score, Model for end-stage Liver Disease score; BMI, body mass index; DM, diabetes mellitus; INR, international normalized ratio; WBC, white blood cells; HR, heart rate; CVP, central venous pressure; EAD, early allograft dysfunction; POD, postoperative day; PS, propensity score; IQR, interquartile; CRP, C-reactive protein.
